# Endothelial Progenitor Cell-Derived Extracellular Vesicles: Potential Therapeutic Application in Tissue Repair and Regeneration

**DOI:** 10.3390/ijms22126375

**Published:** 2021-06-15

**Authors:** Sonia Terriaca, Elena Fiorelli, Maria Giovanna Scioli, Giulia Fabbri, Gabriele Storti, Valerio Cervelli, Augusto Orlandi

**Affiliations:** 1 Department of Biomedicine and Prevention, Anatomic Pathology Institute, University of Rome Tor Vergata, 00133 Rome, Italy; sonia.terriaca@uniroma2.it (S.T.); elena.fiorelli@uniroma2.it (E.F.); scioli@med.uniroma2.it (M.G.S.); fabbrigiulia25@gmail.com (G.F.); 2 Plastic and Reconstructive Surgery, Department of Surgical Sciences, University of Rome Tor Vergata, 00133 Rome, Italy; gabriele.storti@uniroma2.it (G.S.); valeriocervelli@virgilio.it (V.C.)

**Keywords:** endothelial progenitor cells, extracellular vesicles, injury, recipient cells, tissue regeneration

## Abstract

Recently, many studies investigated the role of a specific type of stem cell named the endothelial progenitor cell (EPC) in tissue regeneration and repair. EPCs represent a heterogeneous population of mononuclear cells resident in the adult bone marrow. EPCs can migrate and differentiate in injured sites or act in a paracrine way. Among the EPCs’ secretome, extracellular vesicles (EVs) gained relevance due to their possible use for cell-free biological therapy. They are more biocompatible, less immunogenic, and present a lower oncological risk compared to cell-based options. EVs can efficiently pass the pulmonary filter and deliver to target tissues different molecules, such as micro-RNA, growth factors, cytokines, chemokines, and non-coding RNAs. Their effects are often analogous to their cellular counterparts, and EPC-derived EVs have been tested in vitro and on animal models to treat several medical conditions, including ischemic stroke, myocardial infarction, diabetes, and acute kidney injury. EPC-derived EVs have also been studied for bone, brain, and lung regeneration and as carriers for drug delivery. This review will discuss the pre-clinical evidence regarding EPC-derived EVs in the different disease models and regenerative settings. Moreover, we will discuss the translation of their use into clinical practice and the possible limitations of this process.

## 1. Introduction

In recent years, the use of adult stem cells has progressively been extended to methods and protocols for tissue regeneration and repair. Among stem populations, a particular type of stem cell called “endothelial progenitor cells” (EPCs) seems to be very advantageous [1]. EPCs represent a heterogeneous population of resident mononuclear cells that originate in the bone marrow [1]. They are CD34-positive cells and were isolated for the first time in 1997 by Asahara and coworkers from peripheral blood circulation using magnetic microbeads [2]. Under specific stimuli, such as tissue injury, EPCs mobilize and home to the damaged site through blood circulation [1]. These progenitor cells show some typical stem cell features, such as clonal expansion and angiogenic capability [2]. EPCs are implicated in different processes, including vascular homeostasis, neovascularization, vascular repair, endothelial regeneration, and angiogenesis [3]. EPCs express endothelial antigens such as CD31, von Willebrand factor (vWF), endothelial nitric oxide synthase (eNOS), VE-cadherin, vascular endothelial growth factor receptor 2 (VEGFR2), and can differentiate into mature endothelial cells [4]. Local endothelial differentiation of EPCs favors neoangiogenesis and promotes tissue repair and regeneration [5].

In vitro studies revealed that there are at least two distinct subpopulations of EPCs: early EPCs and late EPCs [6]. Early EPCs appear in culture after 3–5 days and are obtained through a negative selection on fibronectin-coated plates. These cells display a round shape, a slow proliferation rate, and secrete angiogenic factors contributing to neovascularization [6]. In addition, early EPCs show high expression of hematopoietic, monocyte, and endothelial markers and can generate “endothelial cell colony-forming units” in vitro. It has been suggested that early EPCs could derive from hematopoietic stem cells and, for this reason, they are also called “hematopoietic EPCs” [7].

Late EPCs appear after 2–4 weeks in culture and are isolated by positive selection on collagen-coated plates [6]. These cells are elongated cells with a cobblestone-morphology on a monolayer, resembling endothelial cells in vitro and displaying higher proliferative and clonogenic potential when compared with early EPCs. Moreover, late EPCs can form tubular structures such as capillaries in vitro, further supporting their high vasculogenic and angiogenic potential [7]. Late EPCs can be incorporated into an existing endothelium in vivo to form mature vessels by differentiation into mature endothelial cells [7]. Unlike early EPCs, late EPCs do not exhibit hematopoietic and monocyte markers but express endothelial antigens only [8]. In addition, late EPCs generate “endothelial colony-forming cells or ECFCs” in vitro [9], and, for this, they are considered the legitimate endothelial progenitor cells and are also named “non-hematopoietic EPCs” [7].

EPCs release paracrine factors such as growth factors, cytokines, chemokines, and bioactive lipids that influence cell biology in damaged organs [5]. Abdelgawad et al. [7] classified five groups of “EPC-derived factors”: the first group is involved in vasculogenesis, angiogenesis and includes neuropilins, semaphorins and VEGFR1, 2, and 3; the second group contains factors implicated in endothelial cells/EPCs–immune cell interaction, proliferation, migration, survival, apoptosis, angiogenesis, immunogenicity and immune-modulation, such as tumor necrosis factor alpha (TNF-α), tumor necrosis factor receptor 2 (TNFR2/P75), tumor necrosis factor receptor 1 (TNFR1/P55), and tumor necrosis factor-related apoptosis-inducing ligand (TRAIL); the third group of factors promotes the proliferation, survival, migration, and differentiation of vascular stems/progenitors and includes platelet-derived growth factor (PDGF), bone morphogenetic protein (BMP), wnt signaling, vascular endothelial growth factor (VEGF), transforming growth factor beta (TGF β), basic fibroblast growth factor (FGF2), insulin-like growth factor-1 (IFG-1), and epidermal growth factor (EGF); the fourth group comprises small molecules of non-coding single-stranded RNAs (miRNA, lncRNA, rRNA, tRNA) with regulatory activities; the fifth group contains factors involved in the internalization of ligands, endocytosis, migratory and/or invasive capacity and motility, such as urokinase plasminogen activator (uPA), urokinase plasminogen activator receptor (uPAR), urokinase plasminogen activator receptor associated protein (uPARAP), tissue-type plasminogen activator (tPA), Neuropilins, VEGFR1, 2 and 3, platelet endothelial cell adhesion molecule (PECAM-1), intercellular adhesion molecule 1 (ICAM-1), VE-cadherin, ephrins and epidermal growth factor-like protein 7 (EGFL7) [7]. EPCs, like other types of stem cells and tumor cells, can secrete their paracrine bioactive factors through the release of extracellular vesicles (EVs) [10]. The study of EV biology is an expanding research field because of EVs’ potential as therapeutic tools for treating various diseases, including neurodegeneration, cardiovascular dysfunction, and cancer. Although their classification is continuously evolving, EVs can be divided into three main types: exosomes, microvesicles, and apoptotic bodies [11]. Exosomes have a size between 50 and 100 nm and originate from the endosomal system. They are a homogeneous population with a cup-like shape [11]. Microvesicles have a 100–1000 nm size and are derived from outward blebbing of the plasma membrane [11]. Apoptotic bodies are only released during the last step of apoptosis and have a size between 400 and 1000 nm [11]. Different methods can isolate EVs, and the most common are: ultracentrifugation, ultrafiltration through membranes with pores for different molecular weights, precipitation by polyethylene glycol, and immunoselection by magnetic beads, ELISA or flow cytometry for EV-specific markers such as CD9, CD63, CD81 [12,13]. EVs, released by EPCs, can be incorporated into different types of cells (recipient cells) located in the damaged tissues, stimulating proliferation, differentiation, migration, and angiogenesis [14]. According to the recent findings in the literature, we will discuss potential therapeutic strategies based on the application of EPC-derived EVs to promote tissue regeneration in different diseases.

## 2. EV Isolation and Characterization

Different methods for EV isolation are described in the literature [15]. The most common method is ultracentrifugation that exploits the separation of particles according to their buoyant density. The efficiency of EV isolation by centrifugation depends on many factors, such as acceleration, type of rotor, and its characteristics and the viscosity of the sample [16]. Ultracentrifugation has different advantages, including the possibility to work with large volumes and low cost [16]. Some disadvantages of this method are contaminants in the EV preparation and its long duration that limit both its effectiveness and its use in clinical studies and in diagnostics [17].

Other methods for EV isolation, based on the separation according to their size, are ultrafiltration and gel filtration [18,19]. In the ultrafiltration, filters with pore diameters between 0.22 and 0.1 μm are used. However, contamination with non-EV proteins is possible [20].

Gel filtration is a standard method used for the separation of biopolymers, and itis also applicable for the separation of EVs [21]. However, a pretreatment and concentration of EV samples are necessary to eliminate impurities such as proteins and lipoproteins [22]. Another method for EV isolation is the use of different molecules, such as PEG solutions, protamine, sodium acetate, and organic solvents that allow for EV aggregation and precipitation through electrostatic interactions with the hydrophobic lipid bilayer of the EV plasma membrane, thus neutralizing EV surface charge [15]. The advantages of EV precipitation are the amount, quality, and size of EVs isolated through a quick and straightforward method, which are comparable to those obtained by ultracentrifugation and ultrafiltration [23]. The disadvantage is the contamination of the sample with proteins, lipoproteins, nucleoproteins, viral particles, and molecules of biopolymers, which could interfere with further sample analysis [23,24]. Other methods exploit the interaction of specific molecules such as antibodies, lectins, and lipid-binding proteins with lipids, proteins, and polysaccharides exposed on the surface of the EVs [15]. Magnetic beads, highly porous monolithic silica micro-tips, the surface of plastic plates, cellulose filters, and membrane affinity filters are used to capture EVs through the affinity to these proteins. This method makes it possible to obtain EVs uniform in their size, morphology, and protein content without contamination. Despite these advantages, this type of procedure shows some limits, such as costs and difficult handling of large volumes [15].

After isolation, EVs are characterized by the expression of specific markers, including transmembrane proteins (CD63, CD9, CD34, CD55, CD81), transporter-associated proteins (Alix, Tsg101, and ESCRT family), and heat shock proteins (HSP70) [25]. Their biochemical composition can be analyzed through immunostaining, immunoblotting, flow cytometry, and proteomic analysis [26]. Regarding the physical analysis of EVs, different methods, such as transmission electron microscopy and scanning electron microscopy, are helpful to evaluate the morphology of EVs [27]. In addition, to measure EV size and concentration, nanoparticle tracking analysis, dynamic light scattering, and Tunable resistive pulse sensing can also be used [27].

## 3. The Role of EPC-Derived EVs in Tissue Repair and Regeneration

Paracrine communication through EVs has been shown in various physiological and pathological processes [28]. As mentioned above, EVs carry various factors, particularly miRNAs and lncRNAs, which regulate the gene expression of “recipient cells,” influencing different biological processes, such as angiogenesis, proliferation, and apoptosis [14]. EPCs have been extensively considered a possible “cell therapy” to promote tissue repair in recent years. However, because of different concerns regarding the need for a consistent cell source with a stable phenotype and biological activity, infusional toxicity, cellular rejection, ectopic tissue formation, a possible tumorigenic activity, and ethical issues, their use in the clinical practice has been hitherto rare.

**Figure 1:**
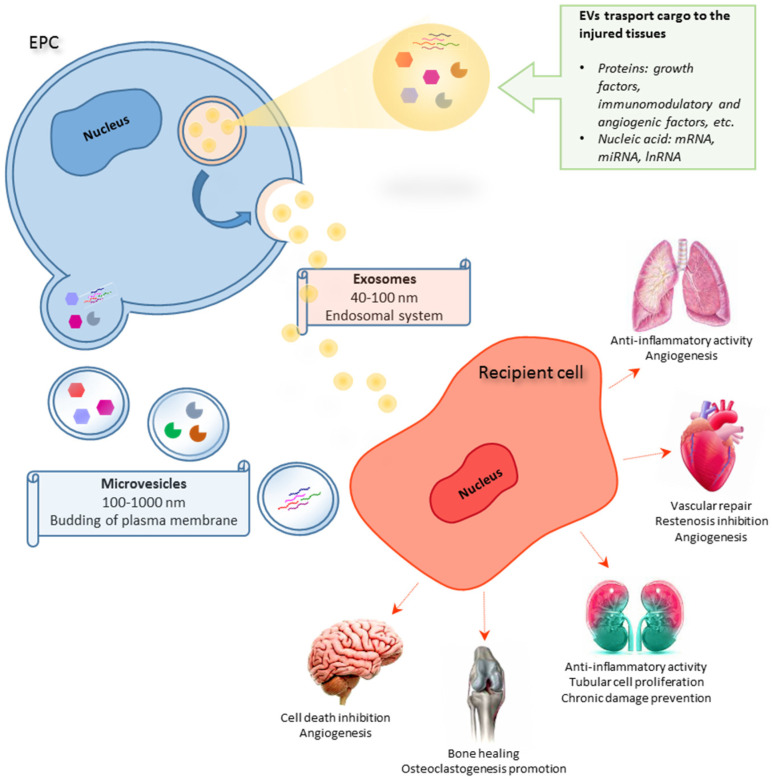
Schematic representation of *EPC*-derived *EV* activities on different target organs.

Consequently, cell-free therapies represent the new frontier in the development of innovative therapeutic approaches [29]. EPC-derived EVs (EPC-EVs) show considerable advantages over their cellular counterparts due to lower immunogenicity, higher safety and stability, and the inability to directly form tumors. They present an innate ability to transport genetic material to target tissues without getting clogged in the lung microvasculature and protecting their cargo from extracellular degradation [30]. For these reasons, EPC-EVs represent promising candidates for cell-free therapies and innovative drug delivery systems in treating different pathologies, such as bone diseases, brain damage, vascular diseases, myocardial infarction, diabetes complications, kidney, and lung injury (Figure 1).

### 3.1. EPC-Derived EVs in Cardiovascular Repair

Cardiovascular disease (CVD) is one of the leading causes of death worldwide [31]. Several pieces of evidence suggest that EPCs are efficacious in promoting neovascularization and tissue repair in pathological conditions such as carotid artery injury, atherosclerosis, thrombosis, and cardiac fibrosis [32]. In particular, some studies proposed EPC-derived exosomes and microvesicles as potential biomedical tools in cell-free cardiovascular therapeutics [33,34]. Percutaneous transluminal angioplasty and stenting treatments, employed in various CVDs, such as coronary artery disease, peripheral artery disease, and carotid artery stenosis, can induce neointima formation and consequently in-stent restenosis [35,36]. Kong et al. [37] investigated the effect of exosomes in a model of restenosis after angioplasty and stenting. In particular, exosomes from embryonic aorta-derived EPCs were injected into the rat carotid artery model after balloon injury. The results showed that the intimal to medial area ratio and the re-endothelialization area were higher in the exosome group than in the control group. In addition, smooth muscle cell proliferation was reduced in the exosome group compared to the controls [37]. So, EPC-derived exosomes could inhibit neointimal hyperplasia by promoting re-endothelialization and suppressing the proliferation and migration of smooth muscle cells.

It has also been reported that microvesicles play an essential role in the initiation, progression, and development of different CVD, such as atherosclerosis, representing a valid therapeutic approach [38,39]. Alexandru et al. [40] evaluated the biological activity and functional role of total and EPC-selected microvesicles (MVs), isolated from the peripheral blood of healthy hamsters in a group of hypertensive hyperlipidemic hamsters. A monthly injection of total and EPC-selected MVs significantly reduced atherosclerosis through the attenuation of dyslipidemia, hypertension, and circulating cytokine/chemokine levels, as well as the structural and functional remodeling of arterial and left ventricular walls. In addition, total and EPC-selected MV treatment increased the number of circulating late EPCs promoting neo-endothelium formation. The authors demonstrated that both total and EPC-selected MVs contained miRNAs, such as miR-223, miR-21, miR-126, and miR-146a, that are transferred to circulating late EPCs protecting them against atherosclerotic injury [40]. These findings support the use of this novel therapeutic strategy against neointima formation during atherosclerosis processes.

The regenerative role of EPC-EVs has also been studied in chronic heart failure following myocardial infarction. In the literature, it has been reported that EPCs and EPC-EVs appear to affect cardiac function [41] positively. In particular, EPC-EV-derived paracrine signaling play an essential role in angiogenesis, proliferation, and cell survival [42]. EVs can be considered a potential cell-free treatment because they can be isolated in large quantities and stored indefinitely [43]. In this regard, Chen et al. [43] proved the beneficial effects of an EPC-EV-mediated treatment in a rat model of myocardial infarction. The authors proved the usefulness of shear-thinning hydrogel application to improve EV delivery and their maintenance in the ischemic area. To that aim, rat EPCs and EPC-EVs were encapsulated or not in shear-thinning hyaluronic acid and injected in the border zone of the infarcted area after the occlusion of the anterior descending coronary artery. The results demonstrated that EPC and EPC-EV injection ameliorated vascular geometry, proliferation, and hemodynamic function compared to the control [43]. This work highlighted that EPC-EV injection has the same beneficial effects as the EPC treatment.

Various EPC-EV-derived miRNAs can promote cardiac function by stimulating cell growth, metabolism, and angiogenesis [41]. In particular, Lin et al. [44] evidenced miRNAs’ role from EPC-EVs in cardiac fibrosis. Myocardial fibrosis occurs when activated fibroblasts proliferate and secrete excessive ECM components, impairing heart function, and structure [45]. Myocardial fibrosis is also characterized by the endothelial–mesenchymal transition, a process that determines the transformation of endothelial cells into myofibroblasts [46]. Human EPC-EVs can promote the expression of endothelial markers in human cardiac fibroblasts favoring the mesenchymal–endothelial transition [47]. Some works have demonstrated that miRNAs are critical regulators of cardiac fibroblast activation [48,49] and are likely involved in the mesenchymal–endothelial transition [45]. Based on these data, Lin et al. [44] focused on EPC-EVs and their effects on myocardial fibrosis and mesenchymal–endothelial transition. Human EPCs were isolated from peripheral blood and subjected to hypoxia/reoxygenation (H/R) to increase mesenchymal–endothelial transition. Rat cardiac fibroblasts cultured with EVs derived from H/R-treated EPCs showed a significant up-regulation of miR-133 and increased expression of endothelial markers compared to those treated with EVs from normoxic EPCs.

Conversely, the inhibition of miR-133 in H/R-treated EPCs decreased endothelial marker expression and consequently mesenchymal–endothelial transition in cardiac fibroblasts [44]. Those findings suggest that EPC-EVs could be employed in therapeutic protocols against fibroblast activation and endothelial–mesenchymal transition occurring in cardiac fibrosis. However, further in vivo investigation is needed to confirm these promising in vitro results.

### 3.2. EPC-Derived EVs in the Treatment of Diabetes

Diabetes is a metabolic disease subject to various complications, such as ulcers, atherosclerosis, and stroke [50]. Diabetic cutaneous ulcers are difficult to treat because they result from the combination of vascular impairment, neuropathy, and infections [50]. Indeed, patients with ulcers have a significantly lower quality of life and life expectancy. For this reason, efficient treatments and therapies are essential. It has been reported that EPC-derived exosomes facilitate skin wound healing by positively modulating endothelial cell function [51]. Xu et al. [52] investigated the potential role and regulatory mechanisms of miRNAs from EPC-derived exosomes in a mouse model of wound healing. They found that exosomes isolated from murine bone-marrowEPCs expressed several miRNAs’, in particular miRNA-221-3p. The administration of exosomes or miR-221-3p alone accelerated skin wound healing in both control and diabetic mice by increasing the expression of angiogenic factors such as VEGF, CD31, and the proliferation marker Ki67 [52]. Those results underline the role of EPC-exosomes, especially the central function of miRNA-221-3p, suggesting new potential approaches to the clinical treatment of diabetic skin wounds.

A significant complication of diabetes is atherosclerosis, characterized by vascular smooth muscle cell proliferation, inflammation, and endothelial damage [53]. After an endothelial injury, EPC-EVs can restore endothelial functions by releasing their content [54]. Bai et al. [55] analyzed the miRNA content in the exosomes from EPCs (from wild-type mice bone marrow) and found a high expression of miR-21a-5p, miR-222-3p, miR-221-3p, miR-155-5p, and miR-29a-3p. The authors used a mouse model of diabetic atherosclerosis (i.e., db/db mice fed with a high-fat diet), in which the exosomes from wild-type mice EPCs or NIH3T3 cell line (as controls) were injected. The treatment with EPC-derived exosomes significantly reduced the production of atherosclerotic plaques and inflammatory factors compared to diabetic mice injected with exosomes from NIH3T3. In addition, the endothelium-dependent contractility of the thoracic aorta of mice treated with EPC-derived exosomes was significantly improved [55]. Those data documented that EPC-derived exosomes could ameliorate endothelial functions in atherosclerotic diabetic patients by releasing specific regulatory miRNAs.

### 3.3. EPC-Derived EVs in Kidney Pathology

Glomerulonephritis is a renal disease in which glomerular inflammation is determined by a wide range of immune-mediated disorders that can lead to acute kidney injury or gradually progress to chronic renal failure [56]. Experimental models for glomerulonephritis can be obtained by injecting antibodies against Thy-1.1, a protein expressed on the membrane surface of renal mesangial cells, provoking an early invasion of platelets, polymorphonuclear leukocytes, and monocytes into glomeruli [57]. Then, complement-dependent mesangiolysis appears with a consequent rebound proliferation of the activated mesangial cells [57]. The latter causes the accumulation of mesangial matrix and, therefore, a massive mesangial injury leading to proteinuria [58]. Furthermore, the glomerular infiltration of monocytes-macrophages also causes endothelial cell loss [59]. Several studies highlighted the potential role of EPCs in the repair of injured glomeruli [60,61]. Cantaluppi et al. [62] reported the effects of EPC-EV treatment in an experimental model of glomerulonephritis. EVs isolated from human EPCs were injected into a rat model of mesangiolytic anti-Thy1.1 glomerulonephritis. EVs localized into the injured glomeruli and inhibited mesangial cell activation, leukocyte infiltration, and apoptosis, as well as proteinuria. In addition, this treatment improved renal function. The treatment with RNase (1U/mL) significantly inhibited the protective action of EVs against glomerulonephritis, suggesting a possible involvement of EV-derived mRNAs in the molecular mechanisms underlying this process. EPC-EVs’ beneficial effects were also analyzed in vitro on cultured rat mesangial cells incubated with anti-Thy1.1 antibody and rat or human serum as a source of complement factors. Activated mesangial cells receiving EV-derived mRNAs coded for Factor H, CD55, and CD59 and counteracted mesangial cell apoptosis and C5b-9/C3 deposition [62]. Therefore, EPC-EVs could exert a protective effect in glomerulonephritis patients by inhibiting the activation of mesangial cells and thus preventing acute kidney injury or chronic renal failure.

Acute kidney injury (AKI) is also a common complication occurring during sepsis [63], in which organ dysfunction and the vascular repair process seem to be also regulated by EPCs and EVs [64]. Beneficial effects of microRNA-126-5p and 3p released by EPC-EVs on kidney function have been reported in a mouse model of sepsis [65]. In another study by He et al. [66], the clinical significance and biological functions of EV-derived miR-93-5p were demonstrated in sepsis-induced AKI. A previous study had already reported a crucial role of miR-93-5p and its molecular target, the lysine (K)-specific demethylase 6B (KDM6B), in AKI patients [67]. In addition, KDM6B had already proved to be involved in inflammatory processes [68]. He et al. [66] used an in vitro model in which human tubular epithelial cells (HK2) were treated with lipopolysaccharide (LPS) to induce an inflammatory injury and validate this supposed interaction. They observed that miR-93-5p and histone H3 Lys27 trimethylation (H3K27me3) were downregulated, while KDM6B was upregulated in LPS-treated HK2. The authors confirmed the interaction between miR-93-5p and KDM6B and the positive correlation between KDM6B and TNF- α expression. Indeed, they pointed out that the binding of KDM6B to the TNF-α promoter was induced by LPS treatment and the silencing of KDM6B upregulated H3K27me3 and its inhibitory activity on the TNF-α promoter. The addition of human EPC-EVs to HK2 counteracted inflammation and downregulated KDM6B by transferring miR-93-5p. The downregulation of miR-93-5p, by transfecting EPCs with its inhibitor, no longer contrasted LPS-induced injury leading to an increase in KDM6B expression, NAG (a marker of tubular cell injury) release, and cell apoptosis. In addition, a reduction in HK2 cell number, cell contraction, cell contact, and E-cadherin expression was observed. In a mouse model of AKI, obtained by cecal ligation and puncture, the injection of EPC-EVs counteracted multiple organ injury, inflammation, and apoptosis by regulating the KDM6B/H3K27me3/TNF-α axis. Indeed, the administration of EVs derived from EPCs transfected with miR-93-5p inhibitor could not provide these beneficial effects [66]. Another study by Cantaluppi et al. [69] reported the positive effects of miR-126 and miR-296 released by MVs from human EPCs, in a rat AKI model. The beneficial role of miR-126 and miR-296 from EPC-derived MVs had already been demonstrated in a murine model of hindlimb ischemia, showing neoangiogenesis and tissue repair [70]. In the study by Cantaluppi et al. [69], ischemia-reperfusion-injured rats were treated with intravenous injection of MVs that localized within peritubular capillaries and tubular cells, induced cell proliferation, and reduced apoptosis and leukocyte infiltration. The authors also reported protective effects of MVs against the progression of chronic kidney damage by counteracting capillary rarefaction, glomerulosclerosis, and tubulointerstitial fibrosis. These improvements were reversed by treating EPCs with RNase, nonspecific miRNA depletion, or transfecting EPCs with specific miR-126 and miR-296 antagomirs [69].

All those studies evidenced EPC-EVs’ role in recovering different kidney diseases, such as glomerulonephritis and sepsis, in which vascular damage and inflammation are predominant. In this way, it is possible to reprogram injured resident renal cells to stimulate regeneration.

### 3.4. EPC-Derived EVs in Bone Healing

Bone repair is a complex process that ends with callus formation [71]. However, inadequate blood supply, reduced angiogenesis, insufficient immobilization, and infection can cause bone repair impairment and, consequently, a severe disability [72]. Osteoclasts resorb redundant bone tissue during bone healing [73]. In addition to osteoclasts, osteoblasts and osteocytes are also involved in the process of fracture healing [73]. It has been reported that EPCs stimulate the proliferation and differentiation of osteoclastic precursors [74] and induce new vessel formation [75]. It has been found that miR-124 is a negative regulator of osteoclastic differentiation [76], and the long non-coding RNA MALAT-1 (LncRNA-MALAT1) interferes with the miR-124 binding site on the integrin subunit β1 in non-small cell lung cancer [77]. Based on this evidence, Cui et al. [78] investigated the regulatory role of LncRNA-MALAT1, contained in EPC-EVs, on osteoclastic migration and differentiation. Macrophages and EPCs were extracted from mice bone marrow and umbilical cord blood, respectively, and EPCs/macrophages co-cultures were performed. They found out an increased migration and osteoclastic differentiation of macrophages compared to single cultures. In addition, a higher expression of LncRNA-MALAT1, β1 integrin, and bone resorption N-telopeptides (NTX) and a reduction in miR-124 in co-cultured macrophages have also been reported. The addition of an exosome inhibitor reduced levels of β1 integrin and NTX and macrophage migration, and increased miR-124 expression in co-cultures by blocking the transfer of LncRNA-MALAT1 to macrophages. Transfecting EPCs with LncRNA-MALAT1-targeting siRNA (Exo-siMALAT1) led to similar findings. In addition, in a mouse model of femur fracture, they transplanted macrophages previously treated with Exo-siMALAT1, demonstrating reduced neovascularization and impaired bone healing compared to the control mice transplanted with macrophages previously treated with Exo-scramble siRNA [78]. These data suggest that EPC-EVs can promote bone repair by enhancing osteoclastogenesis via the LncRNA-MALAT1/miR-124 pathway.

Bone marrow stromal cells (BMSCs), such as EPC-EVs, are critical regulators in bone healing because they promote vessel formation and osteoblastic differentiation [79]. Qin and Zhang [80] evaluated the role of EPC-EVs in BMSC osteoblastic proliferation and differentiation. In particular, EPCs were isolated from murine bone marrow, and their conditioned medium (EPC-CM) was collected. Mouse BMSCs were cultured in osteogenic medium (OM) with EPC-CM, EVs isolated from EPC-CM, or EV depleted EPC CM. EVs negatively regulated the osteoblastic differentiation of BMSCs by reducing osteogenic genes and stimulating cell proliferation [80]. Further studies are needed to identify which factor/s released by EPC-EVs are responsible for the molecular mechanisms underlying bone regeneration.

Another promising application of EPC-EVs, in bone repair, is their use as adjuvant treatment in distraction osteogenesis (DO). DO is a surgical procedure employed to correct defects in long bones, and it consists of three phases: application of external fixators, distraction phase to slowly stretch apart the two bone fragments, and finally, new bone part consolidation. Even though this procedure is widely employed to induce neo-osteogenesis, its limitations are the long duration of the consolidation phase and the increased risk of complications [81]. It is well known that neoangiogenesis proceeds parallel to osteogenesis [82]. Consequently, EPC-derived exosomes have been proposed as a novel therapy for DO management due to their pro-angiogenic effects. In this regard, Jia et al. [83] reported the role of EPC-derived exosomes in promoting angiogenesis, thus favoring bone healing during DO. Exosomes were isolated from rat bone marrow EPCs and injected into unilateral tibial bone distraction gaps in a rat model. Local injection of exosomes accelerated bone regeneration by increased vessel density compared to the control group treated only with PBS. The pro-angiogenic properties of exosomes were also assessed in vitro by a wound-healing assay. This assay revealed that the exposure to exosomes significantly enhanced the motility and tube formation of human umbilical cord blood cells (HUVECs) compared to the control. Based on previously reported data [84] that attributed to miR-126 a possible key role in the pro-angiogenic effects of EPC-EVs, Jia et al. treated HUVECs with exosomes isolated from transfected EPCs with miR-126 inhibitor. Inhibition of miR-126 decreased tube formation, Raf, and Erk1/2 expression, while upregulating SPRED1 [83]. This work suggests that EPC-derived exosomes could be promising candidates in DO management due to their ability to promote the proliferation, migration, and angiogenic capacity of endothelial cells.

### 3.5. EPC-Derived EVs in Brain Regeneration

The integrity of brain microvasculature is crucial in order to keep the functionality of the blood–brain barrier [85]. When a brain injury occurs, angiogenesis can restore BBB functionality and stimulate brain recovery [85]. Brain microvascular endothelial cells (BMECs) are the main constituents of the BBB and are essential for neo-angiogenesis after damage [86,87]. Thanks to their paracrine effects, EPC-derived MVs, as well as exosomes, have been reported to stimulate angiogenesis [88]. Zeng et al. [89] set up specific in vitro experiments to demonstrate the positive effects of EPC-derived MVs in promoting angiogenesis in rat BMECs. BMECs, isolated from cortical tissue and treated with MVs derived from rat spleen EPCs, showed an increased proliferation, migration, and tube formation. Although this is a basic in vitro experiment, it suggests an appealing potential application of EPC-derived MVs in treating brain injury.

Being able to cross the BBB and blood–spinal cord barrier, EPC-EVs are promising candidates for the treatment of neurodegenerative disorders such as amyotrophic lateral sclerosis (ALS) [90]. Recently, various therapeutic strategies, based on EPCs, were developed for this fatal disease. In particular, Garbuzova-Davis et al. [91] underlined the positive effects of human EPCs (from bone marrow) in repairing injured blood–spinal cord barrier in ALS mice by restoring capillary structure through the decrease in permeability and the maintenance of astrocyte perivascular end-feet. Besides these promising results, which mechanisms were involved in the brain microvascular restoration by EPCs is yet to be understood. Subsequently, the same research group [92] proposed a cell-free treatment for the endothelial repair of the BBB exposed to the serum from ALS mice. In particular, human EPCs from bone marrow were cultured with ALS plasma before EV isolation. Then, mouse brain endothelial cells were treated with only ALS plasma or with the addition of EVs at different concentrations. The results evidenced that EVs prevented ALS plasma-induced cell death at the optimal concentration of 1 mg/mL through their incorporation into the endothelial cells. EV internalization was possible by β1 integrin binding; indeed, its inhibition blocked EV beneficial effects on ALS plasma-treated endothelial cells [92]. Thus, these encouraging results obtained in vitro suggest EPC-EVs as possible candidates for future clinical therapies in ALS.

### 3.6. EPC-Derived EVs in Lung Repair

Several works have proposed the use of EPCs in lung repair to promote pulmonary vascular growth and maintenance, but they have also highlighted some limitations, such as the lack of EPC attachment to the lesion site and possible pulmonary embolisms [93]. It has been demonstrated that EPC-EVs can overcome those limitations and can be used to reduce pulmonary inflammation and injury, sustaining their clinical application [94]. The administration of rat EPC-derived exosomes restored the in vivo pulmonary integrity of LPS-treated animals in a model of acute lung injury [94]. Rats treated with EPC-derived exosomes showed reduced interstitial edema and thickness of the alveolar wall, fewer hemorrhages, and a smaller number of inflammatory cells compared to the control group. In addition, exosomes stimulated the in vitro proliferation, migration, and tube formation of LPS-treated endothelial cells. The improvement in endothelial function was related to the exosomal transfer of miR-126 that targeted SPRED1, activating RAF/ERK signaling pathways. Indeed, miR-126 knockdown in EPCs prevented the exosome beneficial properties on LPS-treated endothelial cells [94].

Zhou et al. [95] also examined the role of EPC-derived exosomes and miR-126 in acute lung injury. They injected human EPC-derived exosomes in LPS-treated mice that received protection against lung injury, showing a decreased myeloperoxidase activity and lung injury score. In addition, the exosome group displayed a lower protein concentration in the edema as well as decreased levels of cytokines/chemokines and cell number in the bronchoalveolar lavage fluid, suggesting reduced inflammation and permeability. To assess the role of miR-126 in lung protection, they also injected exosomes from murine fibroblasts (NIH3T3), a cell line not expressing miR-126, with no beneficial effects in LPS-treated mice. Then, they transfected human small airway epithelial cells with miR-126-3p mimic or miR-126-5p mimic. Cells overexpressing miR-126-3p showed decreased PIK3R2 (miR-126-3p target) levels and increased tight junction proteins, probably via Akt pathway activation. Similarly, the overexpression of miR-126-5p reduced mRNA levels of HMGB1 and VEGF-A (inflammatory and permeability factors, respectively) and increased tight junction protein levels [95]. This work confirms the beneficial effects of EPC-EVs and their content, particularly miR-126, in lung repair and suggests their therapeutic potential in human pulmonary diseases.

## 4. Modified EPC-Derived EVs as a Therapeutic Strategy in Tissue Repair and Regeneration

Regenerative medicine aims at the functional restoration of damaged and malfunctioning tissues. One of its approaches is based on cell therapies, in which cells are used to repair tissues either directly or through paracrine stimulation [14]. As previously reported, EPCs offer many attractive characteristics that make them potential candidates for cell therapy [96]. Nevertheless, cell-free therapies could offer more advantages because of broader applications and ethical issues [29]. In this regard, EPC-EVs show higher stability, biocompatibility, lower toxicity, and immunogenicity [29]. As mentioned above, EPC-EVs can regulate cell phenotype, migration, communication, proliferation, and differentiation in a paracrine manner, promoting tissue repair and regeneration [14]. EPC-EVs can be internalized into the “recipient” cells via endocytosis or membrane fusion, transferring their content, including proteins, lipids, mRNAs, miRNAs, and lnc-RNAs [97]. According to these findings, many researchers tried to exploit EVs, isolated from transfected EPCs, as carriers to deliver specific factors to reprogram injured recipient cells and promote their regeneration. Based on previously reported data that pointed out the protective role of miR-210 in ischemic stroke, Ma et al. [84] studied the effects of miR-210-loaded exosomes on hypoxia/reoxygenation (H/R)-injured human endothelial cells in vitro. Human EPCs were transfected with miR-210 mimics and exosomes isolated. The treatment with miR-210-loaded exosomes on the H/R-injured endothelial cells significantly reduced apoptosis, ROS production, and angiogenic dysfunction. Moreover, miR-210-loaded exosomes counteracted mitochondrial dysfunction induced by H/R injury by decreasing fragmentation, MMP, and ATP levels [84]. Therefore, the loading of miR-210 into EPC-derived exosomes could be used to contrast the mitochondrial dysfunction of endothelial cells in ischemic stroke.

The same research group also explored the effects of loading miR-137 through EPC transfection in ischemic stroke [98]. Previous studies had already reported the crucial role of miR-137 in neuronal development and maturation [99,100], EPC proliferation, and angiogenesis in mouse cerebral ischemic stroke [101]. Based on those findings, Li et al. [98] investigated the effects of miR-137-loaded exosomes on the oxyhemoglobin (oxyHb)-injured human neuroblastoma cell line SH-SY5Y. They showed that miR-137 had decreased in SH-SY5Y after oxyHb injury, and the treatment with miR-137-loaded exosomes restored its levels. In addition, cells exposed to miR-137-loaded exosomes displayed a reduced apoptotic rate as well as a decrease in MMP, ROS, and ATP levels. Moreover, SH-SY5Y cells were also protected against lipid peroxidation, iron overload, the degradation of glutathione, and the activation of the COX2/PGE2 pathway [98] that characterize brain injury [102,103]. Therefore, the treatment with miR-137-loaded exosomes could protect neurons against apoptosis and mitochondrial dysfunction in stroke patients.

Ischemic stroke is a potential complication of diabetes [104]. Therefore, Wang et al. [105] investigated the effects of EPC-derived exosomes enriched with miR-126 in a mouse model of diabetic stroke in terms of neurological recovery. MiR-126 had already proved protective against ischemia/reperfusion-induced injury in the hindlimb and kidney [69,70]. Wang et al. [105] transfected mouse EPCs with miR-126 mimics, and then exosomes were isolated. After the ischemic stroke, diabetic mice were intravenously injected with miR-126-enriched exosomes. They found that exosomes localized in brain endothelial cells, neurons, astrocytes, and microglia in the peri-infarct area.

Furthermore, it was demonstrated that exosomes enriched with miR-126 were more effective in reducing infarct size and increasing cerebral blood flow, microvascular density, angiogenesis, neurogenesis, and neurological functional recovery compared to mice administered with non-enriched exosomes. They found downregulation of cleaved caspase-3 and upregulation of VEGFR2 [105]. These results indicate that the enrichment of miR-126 improved the therapeutic efficacy of EPC-derived exosomes in diabetic ischemic stroke by protecting the brain from acute injury. Yi et al. [106] tested the effects of exosomes derived from osteocalcin (OCN)-overexpressing EPCs in endothelial dysfunction. The involvement of OCN had already been found in cardiovascular disease pathogenesis [107], being present at lower levels in the serum of atherosclerotic patients compared to healthy subjects [108,109]. In this regard, Yi et al. [106] transfected rat EPCs with OCN and isolated exosomes. Then, rat aorta endothelial cells (RAOECs) were incubated with OCN-loaded exosomes that promote cell proliferation, migration, and tube formation more than exosomes from not transfected EPCs. These beneficial effects of OCN were likely due to its binding with OCN receptor G protein-coupled receptor family C group 6 member A (GPRC6A) in RAOECs; indeed, their transfection with GPRC6A siRNA blocked OCN activity [106]. From these results, it is possible to hypothesize that OCN-overexpressing-exosomes could be employed to treat endothelial dysfunction in CVD.

Another promising use of modified EPC-EVs has been reported in bone repair. Chen et al. [110] evaluated the effects of MVs from EPCs transfected with miR-126 on osteoblast cells MC3T3-E1. In particular, EPCs from human bone marrow mononuclear cells were transfected or not with miR-126, and the isolated MVs were incubated with osteoblast cells. MVs from not transfected EPCs reduced MC3T3-E1 apoptosis promoting proliferation and migration via ERK1/2-Bcl-2 activation; these effects were enhanced by the miR-126 enrichment [110]. Thus, the combination between EPC-derived MVs treatment and miR-126 enrichment might provide a novel therapeutic strategy for bone regeneration and fracture healing through the stimulation of osteoblastic proliferation. A summarized scheme of all mentioned EPC-EV experimental strategies is reported in Table 1.

## 5. Challenges and Future Perspective

Ongoing clinical trials use exosomes from mesenchymal stem cells to treat diabetes, chronic kidney disease, macular degeneration, cancer, ischemic stroke, and lately COVID-19 [30,111]. Beyond the proven beneficial outcomes, no adverse effects have been reported so far [111,112]. However, the clinical use of EVs is limited because the translation of EV-based therapies to humans requires coping with several issues. Cell culture conditions in physiologically relevant environments, optimal EV isolation protocols to provide batch uniformity, effective EV dose and administration, and in vivo release kinetics of EVs are some of the main concerns [30]. Moreover, the scalability of manufacturing needs to be accounted for [30]. In addition, the entire manufacturing process, including long-term storage and distribution, must be certified as medical grade according to GMP regulations, in high-quality standards, affecting the economic sustainability of the production process [113].

Above all these aspects, a deeper understanding of EVs’ biogenesis and molecular functions is pivotal for the clinical translation of EVs. However, most studies reported EV composition and their mechanism of action in 2D culture systems [114]. Improving the biomimicry of EVs with 3D culture systems would permit the reproduction of a physiological environment [115,116]. In addition, the large-scale manufacture of EVs is pivotal for clinical demand [117].

Several studies have reported using bioreactor systems to achieve EV scalability, with a significant quantity increase [118,119]. Furthermore, it is necessary to determine whether EVs should be obtained from allogeneic or autologous cells. In the first case, allogeneic EVs are a ready-to-use product; on the contrary, autologous ones might require a considerable amount of time, and some pathological conditions require treatments straight away. The possibility of moving to a cell banking system production, which is conformed to manufacturing regulation standards [113], would bypass donor testing for diseases and permit a mass-production from a single donor reducing the variability [113]. Beyond that, cellular age should be taken into account. In fact, some cells in senescence can secrete much more EVs than younger cells [120].

Artificial EVs have been proposed as a possible solution to overcome scalability and heterogenicity problems. Artificial vesicles are easier to produce on a larger scale and have more homogeneity due to a specific control on each phase of the synthesis procedure; consequently, a clinical translation could be facilitated [121]. Among artificial EVs, we find cell-derived nanovesicles (CDNs), EV-inspired liposomes (EVLs), and biomimetic polymer nanoparticles [121]. CDNs are synthesized by a mechanical extrusion, thus forming cell fragments that end with nanosized vesicle formation [121]. CDNs have several advantages, such as the exhibition of higher circulation times, greater yields, and reduced clearance rate [122]. Moreover, it has also been reported that CDNs, similar to native EVs, can shuttle endogenous cargo to recipient cells [123]. However, it is hard to ensure CDNs’ reproducibility due to the difficultly associated with controlling their final composition. EVs are liposomes that can be synthesized “ad hoc” by presenting different peptides on their surface to bind specific recipient cells [124]. The advantage of EVs is the production of an uncontaminated population [117]. Despite this advantage, liposomes have some limitations, such as low stability, retention, and lack of immunomodulatory properties. Polymeric biomaterials offer higher stability than EVLs [121]. In addition, a new class of biologically inspired polymeric nanoparticles called biomimetic polymer nanoparticles can be able to mimic native EVs [121]. They are synthesized with a polymeric core that gives better structural support and stability, allowing for the encapsulation of different cargos [125,126]. The surface is composed of biological material, such as cell plasma membrane, which confers biocompatible features to these nanoparticles [125,126]. Despite these promising features of artificial EVs, more profound knowledge is still required before their clinical application.

## 6. Conclusions

This review reported the beneficial effects of EVs derived from EPCs on tissue repair and regeneration. The use of EPCs, despite their numerous advantages, still presents some concerns regarding their source and ethical issues. Instead, EPC-EVs have higher stability, biocompatibility, lower toxicity, and immunogenicity. For these reasons, EPC-EVs can represent potential candidates for novel cell-free therapies in managing different diseases, such as CVD, kidney injury, diabetes complications, brain damage, lung injury, and bone defects. EPC-EV treatment has proved to be efficacious in promoting angiogenesis, cell survival, migration, proliferation, differentiation, and inflammation reduction. The regulation of those processes in the target cells is likely due to the uptake of paracrine factors, particularly miRNAs, released by EVs.

Moreover, the use of EVs, loaded or enriched with specific regulatory molecules, is likely to influence certain cell functions. The application of EVs from modified “ad hoc” EPCs as vehicles to deliver regulatory factors to injured recipient cells aims to reprogram target cells, enhancing tissue repair and regeneration. However, a deeper understanding of EV biogenesis and their mechanisms of action in the healing process, as well as an evaluation of EV heterogeneity, target selectivity, and species-specific effects are needed to confirm and optimize the beneficial effects of EPC-EV applications in order to develop potential cell-free therapies for the treatment of different diseases.

## Figures and Tables

**Table 1:** Experimental cell-free strategies based on EPC-derived EVs in the treatment of tissue injuries.

Pathology	Experimental Model	EV Type	EV Isolation	EV Factors	EV Modification	Effects of EVs	Ref.
Restenosis	In-stent restenosis model in rats	Exosomes	Polymer precipitation	Not specified	None	Promotion of EC repair and reduction of SMC migration and proliferation	[37]
Atherosclerosis	Hypertensive- hyperlipidemic hamster model	Microvesicles	Immuno precipitation	miR-10a, miR-21, miR-126 miR-146a	None	Increase in circulating late EPCs and vascular repair	[40]
	Diabetic atherosclerotic model in mice	Exosomes	Polymer precipitation	miR-21a-5p, miR-223-3p, miR-155-5p, miR-29a-3p	None	Reduction in atherosclerotic plaques and inflammatory factors	[55]
Myocardial infarction	Myocardial infarction model in rats	Not specified	Polymer precipitation	Not specified	None	Restoration of vascular structure and enhancement of hemodynamic function	[43]
Myocardial fibrosis	In vitro model of H/R-injured rat fibroblasts	Exosomes	Ultracentrifugation	miR-133	None	Increase in angiogenesis and MEndoT	[44]
Stroke	In vitro H/R-injured human ECs	Exosomes	Ultracentrifugation	miR-210	Loading of miR-210	Increase in angiogenesis, improvement in mitochondrial functions, reduction of apoptosis and ROS level	[84]
	In vitro oxyHb-injured. SH-SY5Y	Exosomes	Ultracentrifugation	miR-137	Loading of miR-137	Prevention of cell apoptosis and mitochondrial dysfunction	[98]
	Diabetic stroke model in mice	Exosomes	Immuno precipitation	miR-126	Enrichment of miR-126	Reduction of the infarcted area, increase in cerebral blood flow, promotion of angiogenesis and neurogenesis	[105]
Wound healing	Diabetic cutaneous ulcers in mice	Exosomes	Polymer precipitation	miR-221-3p	None	Stimulation of angiogenesis and enhancing wound healing	[52]
Glomerulonephritis	Anti-thy1.1 glomerulonephritis model in rats; in vitro anti-thy1.1-treated rat mesangial cells	Not specified	Ultracentrifugation	Factor H, CD55, CD59 mRNA	None	Inhibition of complement-mediated injury; reduction in cell death	[62]
Acute kidney injury	AKI model in mice; in vitro LPS-treated HK2	Not specified	Ultracentrifugation	miR-93-5p	None	Amelioration of multiple organ injury, inflammation and apoptosis	[66]
	AKI model in rats	Microvesicles	Ultracentrifugation	miR-126 and miR-296	None	Tubular cell proliferation, reduction of apoptosis and leukocyte infiltration and prevention of chronic damage	[69]
Bone defects	Femur fracture model in mice; mouse BMMs in vitro	Exosomes	Ultrafiltration	Lnc- MALAT1, ITGB1	None	Promotion of bone healing and osteoclastogenesis	[78]
	Mouse BMSCs	Not specified	Ultracentrifugation	Not specified	None	Inhibition of osteogenesis and increase in BMSC proliferation	[80]
	DO model in rats; in vitro wound healing assay in HUVECs	Exosomes	Ultrafiltration	miR-126	None	Promotion of bone regeneration and angiogenesis and cell proliferation	[83]
	Mouse MC3T3-E1 in vitro	Microvesicles	Ultracentrifugation	miR-126	Enrichment of miR-126	Reduction of apoptosis and increase in proliferation and migration	[110]
Brain damage	Rats BMECs in vitro	Microvesicles	Ultracentrifugation	miR-210	None	Promotion of proliferation, migration and tube formation	[89]
Amyotrophic lateral sclerosis	ALS plasma-treated mBECs	Exosomes	Polymer precipitation	Not specified	None	Prevention of cell death	[92]
Acute lung injury	ALI model in rats; in vitro LPS-injured HUVECs	Exosomes	Ultracentrifugation	miR-126	None	Reduction of interstitial edema, alveolar wall thickness and inflammatory cell number; induction of cell proliferation, migration and tube formation	[94]
	Lung injury model in rats; human SAECs in vitro	Exosomes	Polymer precipitation	miR-126	None	Reduction of inflammation and permeability	[95]
Endothelial dysfunction	RAOECs	Exosomes	Polymer precipitation	OCN	OCN overexpression	Stimulation of angiogenesis	[106]

Abbreviations: H/R, hypoxia/reoxygenation; MEndoT, mesenchymal-epithelial transition; ECs, endothelial cells; RAOECs, rat aorta endothelial cells; OCN, osteocalcin; GPRC6A, G protein-coupled receptor family C group 6 member A; AKI, acute kidney injury; HK, human tubural epithelial cells; IRI, ischemia-reperfusion injury; BMMs, bone marrow derived macrophages; BMSCs, bone marrow stromal cells; DO, distraction osteogenesis; HUVECs, human umbilical vein endothelial cells; MC3T3-E1, mouse osteoblast precursor cell line; BMECs, rats brain microvascular endothelial cells; ALS, amyotrophic lateral sclerosis; mBECs, mouse brain endothelial cells; ALI, acute lung injury; SAECs, small airway epithelial cells; oxyHb, oxyhemoglobin; SH-SY5Y, human neuroblastoma cell line.

## Data Availability

Not applicable.
